# A Rare Case of Metastatic Uveal Melanoma Responding to Immunotherapy

**DOI:** 10.7759/cureus.26146

**Published:** 2022-06-21

**Authors:** Celine A Fadel, Swathi Kanakamedala, Shivang U Danak, Andrew T Johnson

**Affiliations:** 1 Internal Medicine, Northeast Georgia Medical Center Gainesville, Gainesville, USA; 2 Hematology-Oncology, Longstreet Clinic Cancer Center, Gainesville, USA

**Keywords:** intraocular malignancy, class ii genes, gna 11 mutation, immunotherapy, uveal melanoma, metastatic uveal melanoma

## Abstract

Uveal melanoma (UM) is an intraocular malignancy with poor survival rates due to the propensity for metastatic spread. Although treatment options exist for localized disease, there are fewer definitive guidelines for metastatic UM. Treatment involves a personalized approach that entails patient-specific aspects, including tumor genetics. This case highlights the disease course of a 60-year-old male diagnosed with stage IIB right eye choroidal melanoma. Despite successful therapy for localized UM, he developed widespread metastasis. He received dual immunotherapy and was ultimately maintained on a single-agent regimen. His prognosis has surpassed initial prognosis and survival expectations. This case highlights the use of immunotherapy, both dual and single therapy, to treat this rare malignancy and extend overall survival.

## Introduction

Melanomas of the choroid, ciliary body, and the iris of the eye are collectively known as uveal melanomas (UMs). UM is the most common primary intraocular malignancy. It is commonly seen in middle-aged Caucasian males with a median diagnosis between 55 and 60 years of age [1]. The most common site for UM is the choroid [2]. The diagnosis is made by ophthalmologic slit-lamp biomicroscopy. For small tumors, anterior-segment optical coherence tomography (AS-COT) is useful in making the diagnosis by showing high-resolution imaging of the anterior and lateral segments. For large lesions, ultrasound biomicroscopy and AS-COT assist in the visualization of the extent of the tumor in the posterior segment. Fine-needle aspiration biopsy can be utilized to confirm the diagnosis of small lesions [2]. UM commonly presents as painless loss or distortion of vision. Some lesions are asymptomatic and incidentally identified during routine ophthalmic screening [1]. The initial workup involves imaging of the abdomen to look for metastatic lesions due to the approximately 90% propensity of hepatic spread metastases by UM. Because these tumors are F-fluorodeoxyglucose (FDG)-avid, positron emission tomography/computed tomography (PET/CT) is often performed [1]. Treatment is individualized and follows general guidelines and principles from oncologists based on the underlying tumor, which ranges from close serial observation, laser therapy, radiation therapy, to surgery (enucleation) for large tumors [1]. In the case of metastatic UM (MUM), there is an ongoing search to establish an ideal systemic therapy. There is no role of chemotherapy in MUM because it is highly resistant to systemic cytotoxic chemotherapy [1]. Due to the growth of UM in one of the most capillary-rich tissues of the body (choroid), it provokes hematogenous spread. The UM cell lines also strongly synthesize and secrete vascular endothelial growth factor (VEGF) and basic fibroblast growth factor (bFGF) [3]. Increased angiogenesis is noted in cancer, with the growth of blood vessels supplying the nutritional and metabolic demands of tumors [3]. Therefore, inhibiting VEGF targets angiogenesis and inhibits tumor growth. Currently, immunotherapy with immune checkpoint inhibitors (ICIs), such as ipilimumab, is a therapeutic option as it has shown promising results in treating cutaneous malignant melanoma [2]. When looking at the programmed cell death protein-1 (PD-1)/programmed death ligand-1 (PD-L1) immune checkpoint, it was noted to be less upregulated in UM compared to cutaneous melanoma. Therefore, ICI therapy is less effective in MUM [4]. Single-agent therapy ICI has shown low response rates, as noted with a 0% overall response rate (ORR) in one phase 2 trial and a 3.6% ORR in another trial [5]. Here, we present the case of a 60-year-old male who was diagnosed with choroidal melanoma in May 2017, subsequently developing MUM and undergoing treatment with dual immunotherapy.

## Case presentation

A 60-year-old Caucasian male presented to his primary care physician in January 2017 with the chief complaint of blurry vision in his right eye, sinus pressure, and dizziness for three weeks. On the physical examination, he had right hemianopsia. He was referred to ophthalmology and subsequently to ocular oncology for a definitive diagnosis. In May 2017, through fluorescein angiography and biopsy, the following diagnosis was confirmed: stage IIB cT3a cN0 cM0 right eye choroidal melanoma, measuring 16 mm in base by 7.3 mm in thickness via fundoscopic examination. Treatment ensued with plaque radiation therapy (RT), followed by bevacizumab, a VEGF inhibitor, every three to four months. Genetic analysis was performed, noting monosomy 3 and gain of 8q. To exclude metastatic disease, he underwent magnetic resonance imaging (MRI) of the abdomen which showed a small lesion at the dome of the liver, suggestive of hemangioma and less likely a metastatic focus. He continued to follow up with ocular oncology from 2017 to 2019 and showed good response to RT and bevacizumab. The tumor size regressed from 7.3 mm to 7.1 mm in thickness.

**Figure 1:**
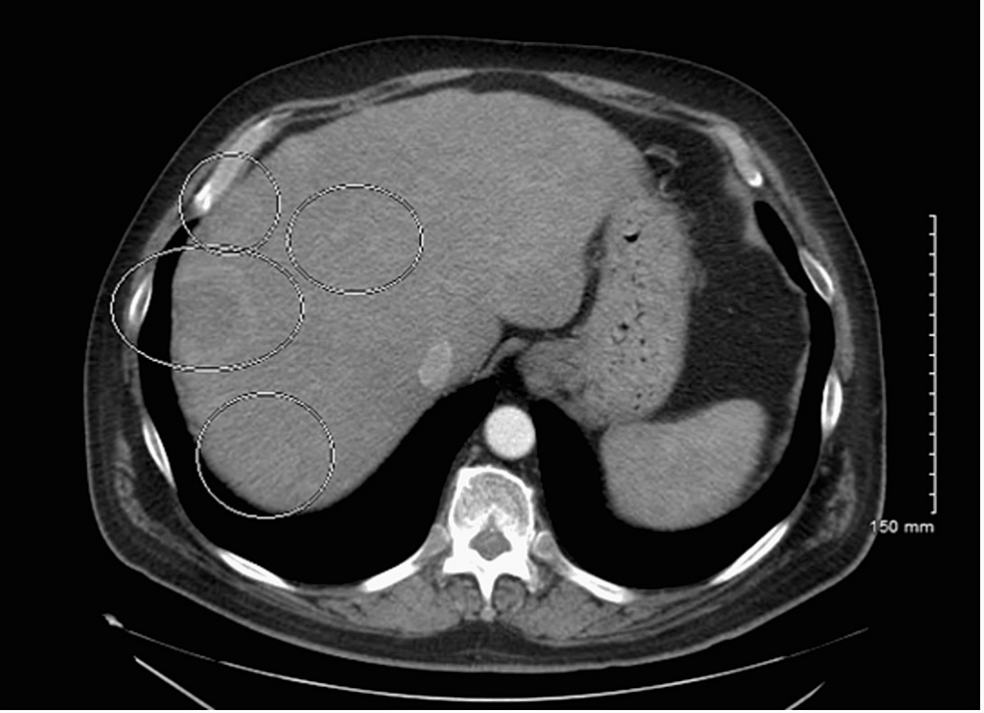
CT of the abdomen and pelvis in May 2020. CT of the abdomen and pelvis in May 2020 demonstrating metastatic liver lesions. CT: computed tomography

In January 2020, he endorsed lower back pain, radiating down his right extremity to his knee. An MRI of the lumbar spine identified a vertebral L3 lesion measuring 2.8 × 3.6 cm with extension into the anterior epidural space. The MRI also revealed additional foci of the T1 marrow signal throughout the lumbar spine and sacrum. A computed tomography (CT) of the chest/abdomen/pelvis (CAP) in May 2020 confirmed findings suggestive of widespread metastatic disease with lesions in the lungs, liver, and adrenal glands (Figure 1). Brain MRI showed no intracranial metastasis. Tissue examination of a CT-guided L3 lesion biopsy revealed malignant cells consistent with melanoma. This prompted palliative RT to the spine from June 6, 2020, to June 15, 2020. Further genetic analysis of the vertebral biopsied tissue identified a GNAQ 11 mutation.

In June 2020, he began dual ICI treatment with ipilimumab and nivolumab. Two months later, treatment was held during hospitalization due to suspicion of immune-mediated pneumonitis. CT CAP in August 2020 showed an interval decrease in the size of the hepatic lesions; more than 30 liver lesions measured less than 2 mm. His pulmonary nodules remained stable. He resumed treatment with ipilimumab and nivolumab.

In September 2020, he was hospitalized for non-severe *Candida *esophagitis, requiring a nasogastric tube and eventual G-tube placement. Immunotherapy was deferred for one month during hospitalization. Due to concern of increased toxicity from dual ICI, the patient resumed single-agent nivolumab in October 2020.

**Figure 2:**
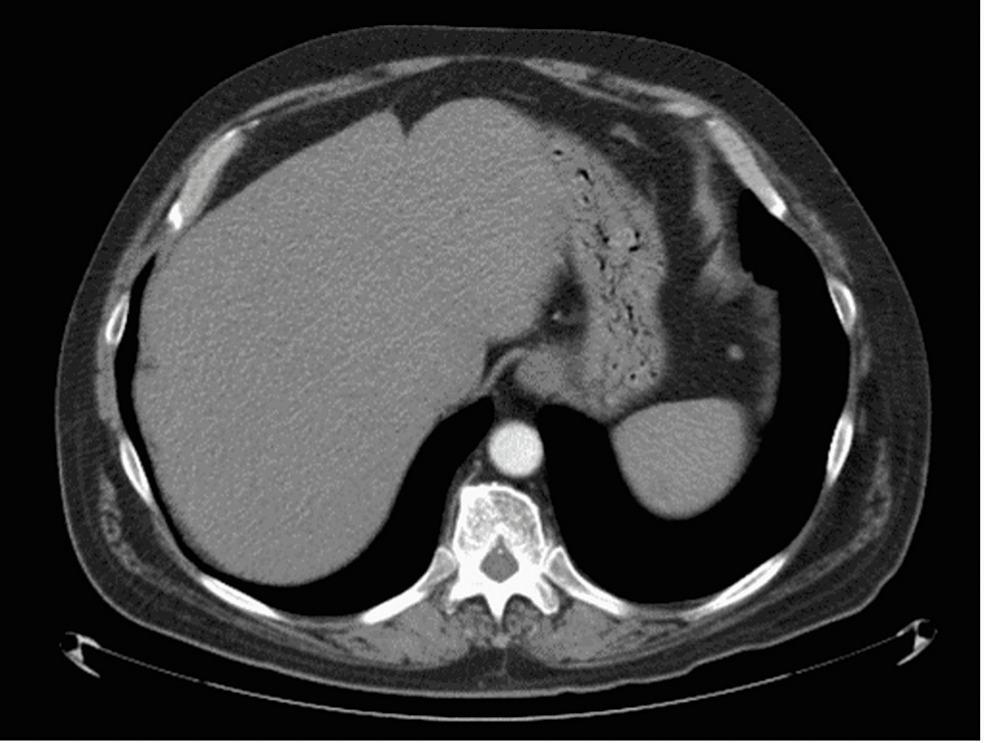
CT of the abdomen and pelvis in February 2022. CT of the abdomen and pelvis in February 2022 demonstrating resolution of previous liver lesions indicating stable disease. CT: computed tomography

From October 2020 to January 2021, serial imaging continued to show a positive response to immunotherapy with complete regression of liver lesions and stable pulmonary nodules. However, in January 2021, CT CAP showed an increase in the size and number of pulmonary nodules within both lungs, concerning for progressive metastatic disease within the chest, whereas there was an interval improvement in metastatic disease within the patient’s liver. From January 2021 to July 2021, he continued to show improvement and resolution of metastatic liver lesions, which remained stable (Figure 2).

The previously noted L3 vertebral tumor regressed. He currently continues immunotherapy with nivolumab. June 2022 marks his third year of ICI treatment. Furthermore, he has been referred to a national clinical trial seeking patients with comparable genetic mutations; he is pending evaluation for possible inclusion in the trial.

## Discussion

As opposed to the high incidence of cutaneous melanoma, UM is considered to be a rare disease with cases noted in 3-5% of the US population [6]. The initial presentation of this patient with a localized choroidal tumor is consistent with the typical UM disease course. Approximately 95% of patients present with uveal involvement, and more than 50% develop metastatic disease [7]. The most common site of metastasis is the liver [3]. Unfortunately, metastasis is associated with poor prognosis, with a five-year survival rate of less than 5% [3].

This patient’s initial tumor was consistent with stage IIB cT3a cN0 cM0 right eye choroidal melanoma. UM is typically staged based on tumor characteristics, with stages ranging from one to four [8]. CT CAP at the time of diagnosis was negative for metastatic disease. The choroidal tumor measured 16 mm in max diameter × 7.3 mm thickness on fundoscopic examination. These measurements are within the National Comprehensive Cancer Network (NCCN) guideline threshold of ≤19 mm base diameter × 2.5-10 mm thickness for options including plaque brachytherapy, particle beam radiation, and enucleation [8]. Intravitreal bevacizumab was administered every three months to prevent elevated levels of VEGFs. This was a prophylactic treatment due to the increase in VEGF with radiotherapy, potentiating radiation maculopathy [9]. Close follow-up ensued, and our patient showed slow regression over time. However, in January 2020, he was diagnosed with widespread metastatic disease.

The treatment options for metastatic melanoma are limited, and the literature does not support any specific agents with superior outcomes [8]. If metastatic disease is confined to the liver, liver-directed therapies can be considered, including regional isolation perfusion, embolization, ablation, resection, and RT [8]. Unfortunately, our patient had widespread metastases. One of the first reports of UM responding to immunotherapy was published by Kottsachade et al., where patients with metastatic disease were treated with pembrolizumab, a PD-L1 inhibitor. Half of the patients had rapidly progressive disease, while others had varying outcomes ranging from stable disease to complete response [10]. On the contrary, a study by Algazi et al. showed no durable remission in patients treated with single-agent PD-1 and PD-L1 antibodies [11]. Literature concerning PD-1 and PD-L1 inhibitors as treatment options is mixed. Other ICI therapies have been studied, including cytotoxic T-lymphocyte-associated antigen antibodies. The first large retrospective study demonstrated a durable response, with average overall survival (OS) of 9.6 months, in patients with MUM treated with ipilimumab [12]. Additionally, two phase 2 studies in 2014 and 2015 utilized ipilimumab for progressive metastatic disease, reporting similar OS of 9.8 and 6.8 months, respectively [13]. It is important to understand that the literature regarding immunotherapy for UM stems from the therapeutic response seen in cutaneous melanoma responding to this treatment. However, there are critical differences between the two malignancies. One such difference is the mutation rates, which are lower in UM. This leads to decreased neoantigens and a smaller chance of immune cell recognition [14].

Recent studies are focusing on the use of dual-agent immunotherapy. The combination has shown an OS of 15 months which is higher than the average OS noted in single-agent therapy [15]. This prompted our decision to use both nivolumab and ipilimumab. Our patient has surpassed the average expected OS; he remains progression-free for two years. Immunogenetics are targetable characteristics for potential treatment options. Our patient was evaluated for high-risk genetic features using a microscopic needle aspirate sample for micro-assay analysis. Genetic expression analysis divides mutations into a binary classification, Class I and Class II genes [16]. Our patient tested positive for monosomy 3 and gain of chromosome 8q, classified as Class II genes [16]. This was performed at the initial diagnosis of localized disease, with localized disease. Class II genes are associated with poor prognosis [2]. This enforced the need for close monitoring of our patient to discover any potential metastatic disease as early as possible. After radiographic confirmation of metastasis, his spinal lesion was sent for genetic analysis, which confirmed a GNA11 mutation; a common oncogene identified in 80% of UM cases [17]. This oncogene activates the protein kinase C (PKC) and mitogen-activated protein kinase pathways inducing cell signaling and proliferation [18]. PKC inhibitors, individually and in combination with MEK inhibitors, have shown a synergistic effect in halting cell proliferation [18]. This prompted our patient’s timely referral to a national clinical trial currently in progress, seeking patients with similar presentations and assessing the use of PKC inhibitors.

## Conclusions

The future of UM is currently under investigation with increasing clinical trials and hypothesized treatment approaches. Although most cases of UM have not shown a clear and consistent response to single-agent immunotherapy, it continues to be under investigation with dual ICI therapies. Based on oncogene findings and immunogenetics, there may be opportunities for targeted therapy with ongoing research. Our case presents a unique observation of successful treatment with ICI with a progression-free survival of almost two years. Our use of clinical and radiographic response, with the addition of genetic analysis, yields a personalized treatment approach beyond expected statistical survival.
